# From Fragile to Resilient Insulation: Synthesis and Characterization of Aramid-Honeycomb Reinforced Silica Aerogel Composite Materials

**DOI:** 10.3390/gels2010001

**Published:** 2015-12-22

**Authors:** Marina Schwan, Matthias Rößler, Barbara Milow, Lorenz Ratke

**Affiliations:** Institute of Materials Research, German Aerospace Center, Linder Hoehe, 51170 Cologne, Germany; matthias.roessler@rwth-aachen.de (M.R.); barbara.milow@dlr.de (B.M.); lorenz.ratke@dlr.de (L.R.)

**Keywords:** fluffy silica aerogels, flexible silica aerogels, honeycomb-composite, thermal insulating, mechanical properties

## Abstract

The production of a new composite material embedding aramid honeycomb materials into nano-porous silica aerogels is studied. Our aim is to improve the poor mechanical strength of silica aerogels by aramid honeycombs without losing the amazing properties of the aerogels like little density and low thermal conductivity. The composite materials were prepared using two formulations of silica aerogels in combination with aramid honeycomb materials of different cell sizes. The silica aerogels are prepared using silicon alkoxides methyltrimethoxysilane and tetraethylorthosilicate as precursors in a two-step acid–base sol–gel process. Shortly in advance of the gelation point, the aramid honeycombs were fluted by the sol, gelation occurred and, after the aging process, the gel bodies were supercritically dried. The properties of the received composite materials are satisfying. Even the thermal conductivities and the densities are a bit higher than for pure aerogels. Most importantly, the mechanical strength is improved by a factor of 2.3 compared to aramid honeycomb materials and by a factor of 10 compared to the two silica aerogels themselves. The composite materials have a good prospective to be used as an impressive insulation material.

## 1. Introduction

Nowadays, incombustible and non-toxic thermal insulation for buildings, industrial applications as well as for the automotive sector are more important than ever. A moderate consumption of energy is highly required to save the natural resources of fossil fuels, reducing the CO_2_ foot print and protecting the world’s climate [1].

Materials like expanded polystyrene, polyurethane foams or mineral wool are well established and already possess a low thermal conductivity and a low density too. However, these materials are only suitable for a limited range of applications. The applicability depends on the thermal stability according to the operating conditions like temperature or mechanical loading range. In addition, a long-term flame resistance, preventing the production of toxic decomposition products is not given too. Even the manufacturing of some is a toxic and energy intensive process.

Aerogels might close this gap. Research on silica aerogels and its thermal properties has already been started by Kistler [2] in the early thirties’ of the last century. They are easy to be prepared by a two-step acid-base sol-gel process followed by a suitable almost shrinkage-free drying procedure. Aerogels and aerogel based materials have a unique combination of physical properties [3]. The morphology of the silica aerogels—with its open-porous nanostructure built of small interconnected particles and small pore sizes (~10 nm) leading to an immense high porosity of up to 99.9%—is responsible for the low density (~100 kg·m^−3^), low thermal conductivity (0.03–0.01 W·(m·K)^−1^), huge internal surface (~1000 m^2^·g^−1^), and low mechanical strength (~50–500 kPa). Especially the impressive low thermal conductivities of silica aerogels down to 0.01 W·(m·K)^−1^ are promising for countless new insulation applications in aeronautics, mobility or the building industry [4]. One key factor that hinders the industrial utilization of aerogels is their fragility, with low Young’s moduli and their characteristic brittleness. These prevent their usage in many fields of application and therefore industrial fabrication of monolithic aerogel for instance in the form of tiles is still not on the way.

Reducing the rigidity and brittleness of aerogels is more than necessary. Several attempts to chemically modify the microstructure have been carried out and lead to soft and mechanically flexible silica aerogels [5,6,7]. For example, Maleki *et al.* proposed polymer-reinforced silica aerogels with compression strength from 11 to 400 kPa and thermal conductivity of 0.039–0.093 W·(m·K)^−1^ [6].

A second focus to improve the mechanical strength of silica aerogels is to fabricate aerogel composite materials. This has been presented by Liao *et al.* in 2012 and Mazraeh-shahi *et al.* in 2014 [8,9]. The combination of aerogels and binders is patented as a successful insulating material with suitable mechanical properties [10], as well as aerogel composites with polyurethane foams [11,12]. One other way to prevent the disintegration of silica aerogels was suggested by Capadona *et al.* The authors could significantly increase the stiffness and strength by crosslinking with isocyanate [13]. Several methods to improve elastic properties are summarized by Meador [14]. In general, a polymer coating on the skeletal nanostructure makes aerogels mechanically stronger [14]. To improve the mechanical properties of silica aerogels, other groups suggest a variety of other approaches, such as incorporation of tungsten disulfide nanotubes [15], chemical vapor deposition treatment with hexamethyldisilazane [16], or polymer-reinforcement allowing ambient drying of silica aerogels [17]. However, organic resorcinol-formaldehyde aerogels and their pyrolized form—carbon aerogels possess also high stiffness. Chen *et al.* investigated a combination of phenolic resin and carbon aerogels. They could increase flexural strength by 18.4% and impact strength by 101% [18].

In this paper, we present a new composite material combining chemically modified—fluffy and low-flexible—silica aerogels with aramid honeycombs for reinforcement. Beside the two types of modified silica aerogels, the honeycomb materials and corresponding honeycomb composite materials were synthesized and characterized. A comparison of the materials is performed with respect to their mechanical and thermal properties as well as their morphology. Their suitability as a light weight super insulating material is proven.

## 2. Results and Discussion

### 2.1. Appearance and Properties of the Primary Materials, Aerogels and Honeycomb Materials

In our study, two different types of aerogels were synthesized and characterized. Due to different precursors we used, the aerogels produced exhibit different haptic and mechanical properties. They both are elastic but with different degrees of deformability. The first type is a methyltrimethoxysilane (MTMS) based silica aerogel, which is highly flexible. It is reversible deformable like a marshmallow. This aerogel, we call in the present study SA1 (silica aerogel 1). Due to its high flexibility, we also call it super-flexible. The second type is an methyltrimethoxysilane- [3-(2,3-Epoxypropoxy)-propyl]-trimethoxysilan (MTMS-GPTMS) based aerogel having lower degree of flexibility. This type is rubber-like and more brittle. We name this aerogel SA2 (silica aerogel 2) or low-flexible by reason of its reduced deformability.

**Figure 1:**
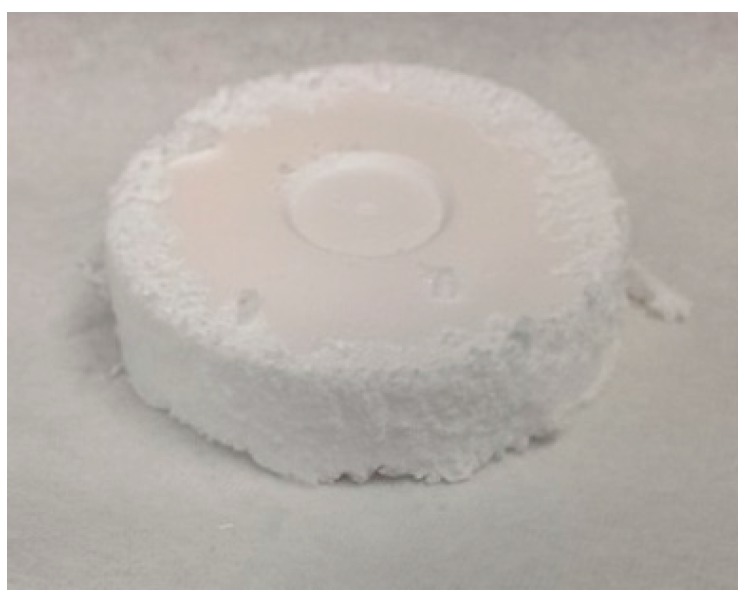
Super-flexible silica aerogel SA1.

**Figure 2:**
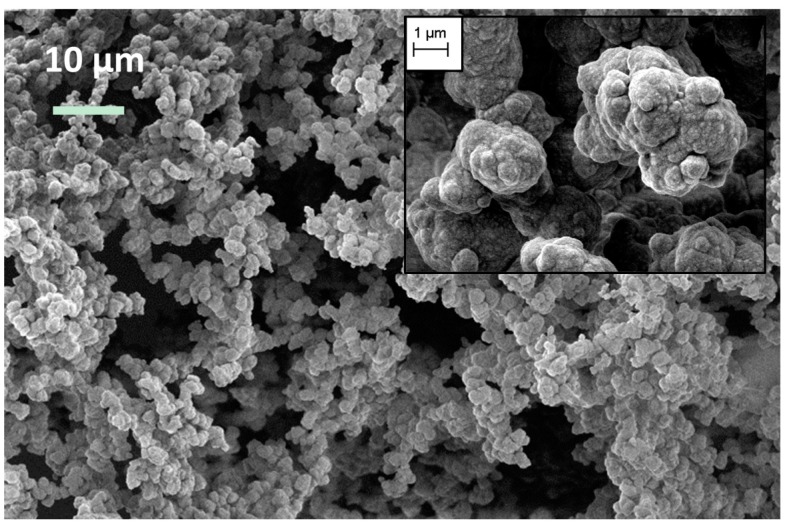
Microstructure of SA1 (magnification of image 1k× and of inset 5k×).

The pure SA1 aerogel, shown in the Figure 1 is plain white and extremely fluffy. When slightly blowing over its surface, aerogel powder comes off and one can hardly feel any counterforce when compressing it by hand. Shrinkage after supercritical drying was below 5%, and is neglected in further considerations. It shows low density (0.037 g·cm^−3^) and low thermal conductivity (0.034 W·(m·K)^−1^), as given in Table 1. Due to the high flexibility, its compressive modulus is only 3 kPa. The SEM image in the Figure 2 shows the microstructure of an SA1 sample with large particles and pore sizes. For flexible aerogels, large pores are responsible for the reversible deformation of the network [19].

**Table 1:** Properties of produced aerogels and honeycombs.

Material	Thermal Conductivity (W·(m·K)^−1^)	Compressive Modulus ^a^ (MPa)	Envelope Density (g·cm^−3^)	Skeletal Density (g·cm^−3^)	Porosity (%)	Mean Pore Size (nm)
Super-flexible aerogel SA1	0.034	0.003	0.037	1.38	97.3	242
Low-flexible aerogel SA2	0.038	0.074	0.092	1.41	93.5	113
Aramid honeycomb C1-3.2-29	0.060 ^b^	0.030 in-plane 10.7 out-of-plane	0.029 ^c^	-	-	-
Aramid honeycomb A10-92-5.2 ^d^	0.07 ^b^	0.086 in-plane 12.2 out-of-plane	0.092 ^b^	-	-	-
Aramid honeycomb C1-6.4-24 ^e^	0.08 ^b^	0.035 in-plane 11.3 out-of-plane	0.024 ^c^	-	-	-

^a^ Due to the fact that aerogels are isotropic, the compression tests on pure aerogels were done only in one direction; ^b^ from data sheet [20]; ^c^ from data sheet [21]; ^d^ the value in [20] is given for cell size 4.8 mm; ^e^ the value in [20] is given for cell size 6.3 mm.

To characterize the porous structure of an aerogel the porosity and mean pore size were calculated. The porosity was determined by using of Equation (1) from envelope *ρ_env_*_._ and skeletal *ρ_skel_*_._ densities.

(1) $$\Phi =\left(1-\frac{\rho_{\text{env}.}}{\rho_{\text{skel}.}}\right)\cdot 100\%$$

The specific pore volume *v_pore_* is given by

(2) $$v_{pore}=\frac{1}{\rho_{env.}}-\frac{1}{\rho_{skel.}}$$

and can be used to calculate the average pore size *d_av_* using [22]

(3) $$d_{av}=\frac{4\cdot v_{pore}}{S_{BET}}$$

The SA1 exhibits high porosity of 97.3% and average pore size of about 242 nm.

**Figure 3:**
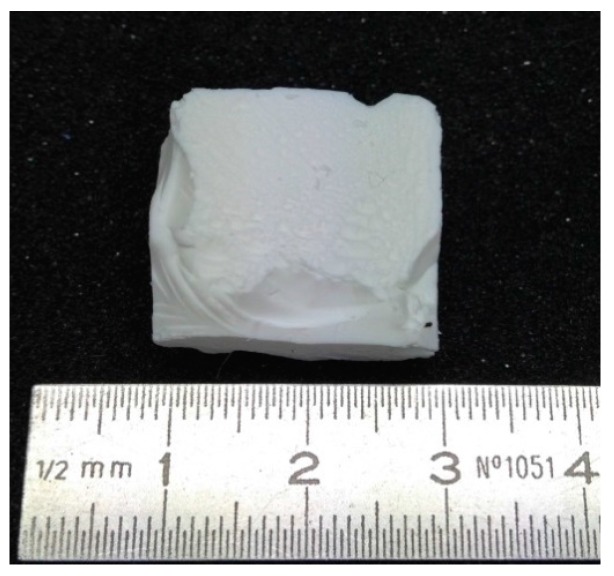
Low-flexible silica aerogel SA2.

**Figure 4:**
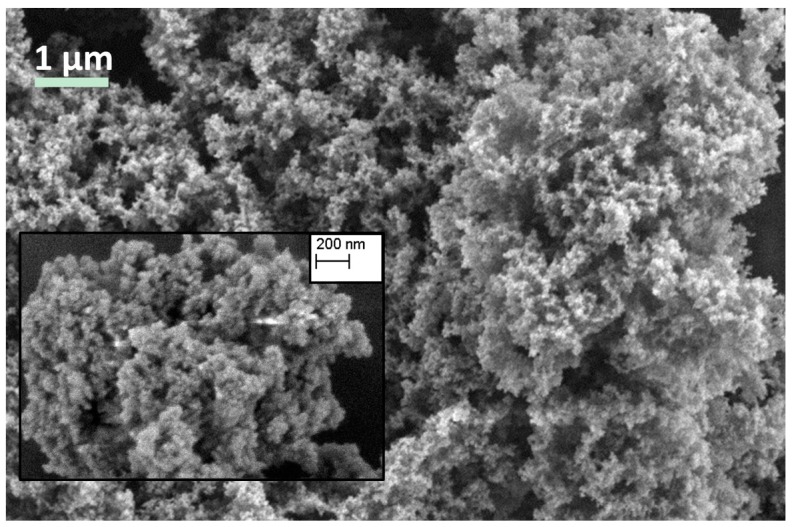
Microstructure of SA2 (magnification of image 10k× and of inset 24k×).

The low-flexible silica aerogel (SA2), shown in Figure 3, is much stiffer in contrast to the SA1. Being white as well, with a haptic like an eraser or rubber, it is still slightly flexible and compressible. Though being significantly more robust than the super-flexible aerogels, the SA2 aerogel is still easy breakable into pieces. Its structure consists of small, interconnected particles and pores in the range of 0.5–1 μm as shown in Figure 4. Compared to SA1 and SA2 aerogels, it appears with a finer structure. The calculation of average pore size confirms that the pores of SA2 are almost two times smaller than SA1. The low-flexible aerogel exhibits higher envelope density (0.092 g·cm^−3^) caused by the higher solid concentration. A slightly higher thermal conductivity (0.038 W·(m·K)^−1^) and an almost 25 times higher compressive modulus compared to SA1 is measured consequentially, as given in Table 1. The porosity of SA2 is slightly lower (93.5%).

**Figure 5:**
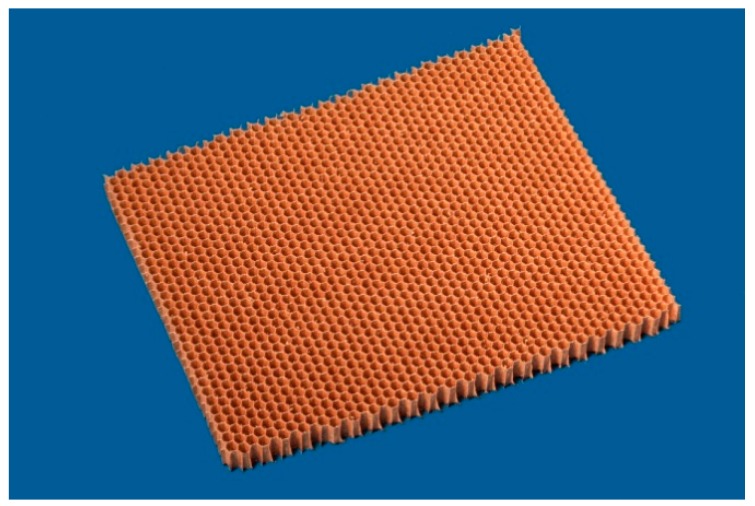
Aramid honeycomb C1-6.4-24 covered with phenolic resin.

The envelope density of aramid honeycombs of type C1, shown in Figure 5, is slightly lower compared to the synthesized aerogels, SA1 and SA2. The envelope density of honeycombs type A10-92-5.2 is similar to SA2 aerogels and much higher than of SA1.

Their thermal conductivity is almost two times higher, due to higher heat transfer via the solid and gaseous phases. The aramid fibers form dense walls, with a density of 1.44 g·cm^−3^ being much higher than that of aerogels [23]. Since the heat transfer via the solid backbone is directly proportional to the density of backbone material, the thermal conductivity of aramid is higher. The heat transfer via gaseous phase depends, amongst other parameters, on the pore dimension. In the cells of 3.2–6.4 mm, the diffusive and convective heat transfer is predominant and leads also to a high thermal conductivity [4]. Filling of cells with aerogel should decrease the heat transfer and lead to better insulating materials.

As expected, the stiffness of aramid honeycomb material is significantly higher than that of aerogels. The honeycomb materials are highly resilient. After releasing a load, they spring back to their initial size and shape. The honeycomb A10-92-5.2 shows the highest density and the highest compressive modulus in both directions. The properties of C1 type honeycombs look similar.

### 2.2. Appearance and Properties of the Aerogel-Honeycomb-Composite Materials

**Figure 6:**
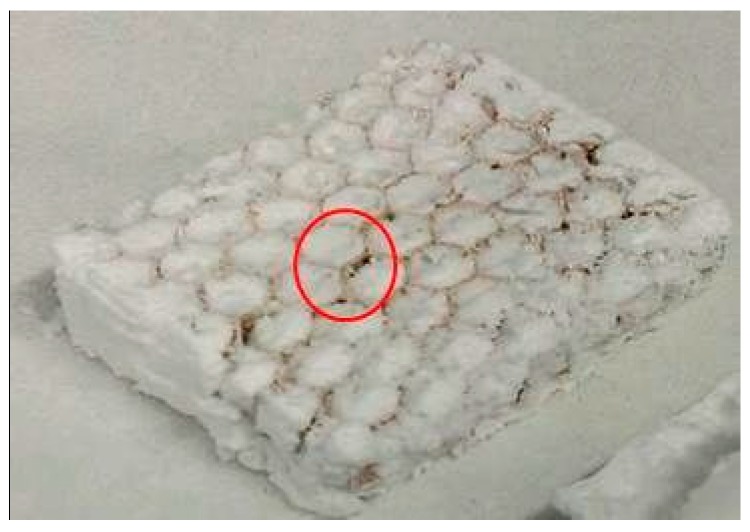
Super-flexible silica aerogel (SA1) with aramid honeycombs A10-92-5.2.

**Figure 7:**
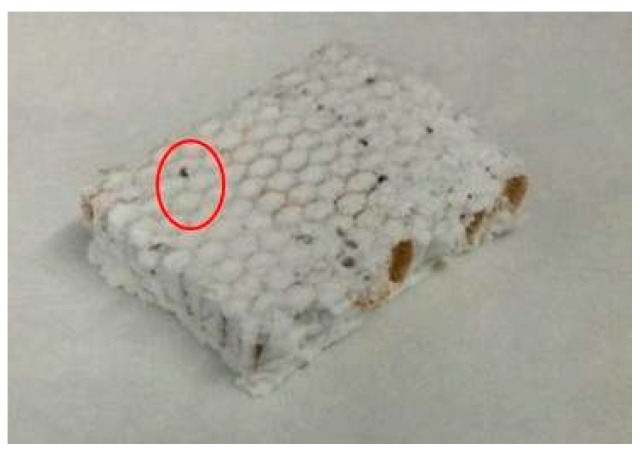
Super-flexible silica aerogel (SA1) with aramid honeycombs C1-3.2-29.

**Figure 8:**
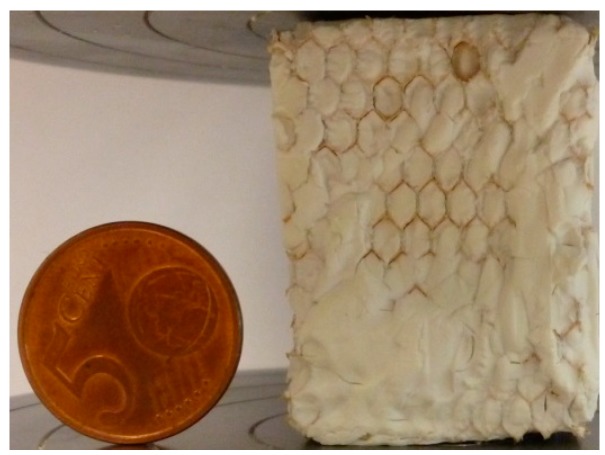
Low-flexible silica aerogel (SA2) with aramid honeycombs C1-3.2-29.

**Figure 9:**
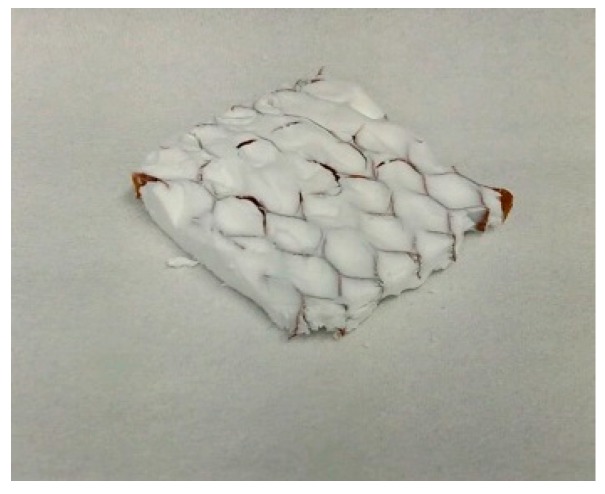
Low-flexible silica aerogel (SA2) with aramid honeycombs C1-6.4-24.

**Figure 10:**
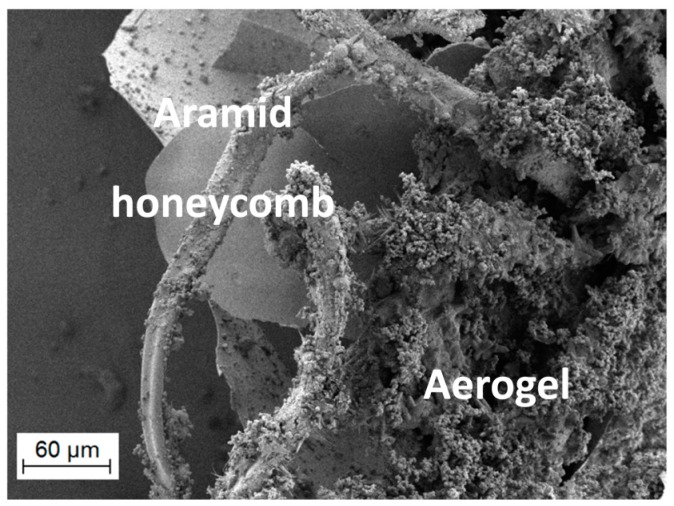
SEM image (magnification 208×) of a cut-off cross-section of the SA1 aerogel-honeycomb composite, showing aramid-fibers, covered with good adhering aerogel particles.

**Figure 11:**
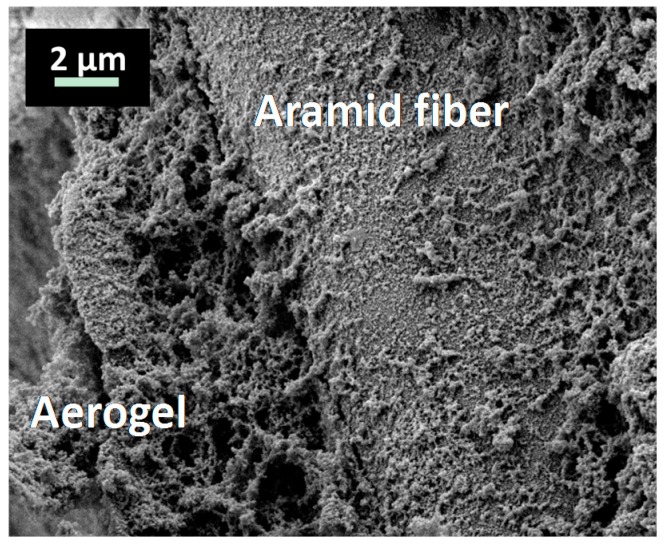
Adhesion of SA2 aerogel particles on aramid fiber (magnification 4.0k×).

The composite materials, depicted in Figure 6 and Figure 7, containing SA1 aerogel show sound adhesion on the honeycomb and surround it thoroughly without any cracks. Some small pores are visible on the surface caused by formation of air bubbles during sol-gel synthesis. These holes (encircled) could negatively affect the composite and cause a weakening. Figure 8 and Figure 9 depict the composites with low-flexible SA2 aerogels. Without any defects inside the material, the samples exhibit good adhesion. The firm contact between aerogels and honeycomb material was additionally approved by SEM. Figure 10 and Figure 11 show both materials: honeycombs and aerogels. One can see aramid fibers covered with aerogel particles. This confirms a continuous, firm contact between the two materials.

### 2.3. Thermal Properties

As already mentioned, heat in solids is transferred via different mechanisms [24,25,26,27]. The composites with higher density should transfer heat faster through their solid network. The density of the composites produced depends on two parameters. First, SA2 aerogels have higher densities, therefore the composites using SA2 aerogel will have a higher density too. Second, the higher the volume fraction of the honeycomb, the higher the envelope density. Composites with honeycomb type C1-3.2-29 (with the smallest cell size) consist of the highest amount of aramid (volume fraction: 7.3 vol.-%) as shown in the Table 2. On the other hand, the higher the volume fraction of the aerogel, the lower the thermal conductivity of composites should be.

We calculated the theoretical thermal conductivity with the rule of mixture

(4) $$\lambda_{eff}=\lambda_{Aerogel}\cdot \Phi_{Aerogel}+\lambda_{Honeycomb}\cdot \Phi_{Honeycomb}$$

where *λ* denotes the thermal conductivities and *Φ* the volume fraction of components. The results are given in the Table 2.

**Table 2:** Properties of produced honeycomb.

Sample	Aerogel (volume %)	Honeycomb (volume %)	Envelope Density (g·cm^−3^)	Theoretical Thermal Conductivity *λ_eff_* (W·(m·K)^−1^)	Measured Thermal Conductivity (W·(m·K)^−1^)
SA1 C1-3.2-29	92.7	7.3	0.069	0.036	0.038
SA1 A10-92-5.2	95.0	5.0	0.062	0.036	0.039
SA1 C1-6.4-24	96.2	3.8	0.073	0.036	0.036
SA2 C1-3.2-29	92.7	7.3	0.091	0.040	0.044
SA2 A10-92-5.2	95.0	5.0	0.092	0.040	0.044
SA2 C1-6.4-24	96.2	3.8	0.091	0.040	0.039

Because of the honeycomb structure, the volume fraction of the filling is over 90%. It depends only on the pores dimension of the honeycomb materials used. As expected, the thermal conductivities calculated for composites are equal, even with different aerogel amounts and different cell sizes of the honeycomb materials. Higher conductivity of SA2 aerogels affects the conductivity of composites, which is slightly higher.

Compared to theoretical values, the thermal conductivities measured for several samples are higher. The differences are negligible and average about 2%–5%. In general, the conductivity in comparison to the honeycomb itself (0.06–0.08 W·(m·K)^−1^) was substantially decreased. With a larger cell diameter, the volume and therefore the mass fraction of the aerogel increase, which results in a decrease of thermal conductivity for the composite material. The best results could be achieved with SA1 aerogel and honeycombs C1-6.4-24. Here, the thermal conductivity could be successfully reduced by 40 percent in comparison to the honeycomb material itself.

### 2.4. Mechanical Properties

Important requirements for insulating materials are a sufficient stiffness, a suitable loading capacity and, in addition, a certain flexibility. Flexible insulation can guarantee a perfect contact between the insulated surface and the insulating material, so that no air or other fluids can flow in-between. The extremely soft flexible aerogels satisfy this requirement if they are reinforced e.g., by flexible honeycomb materials.

**Figure 12:**
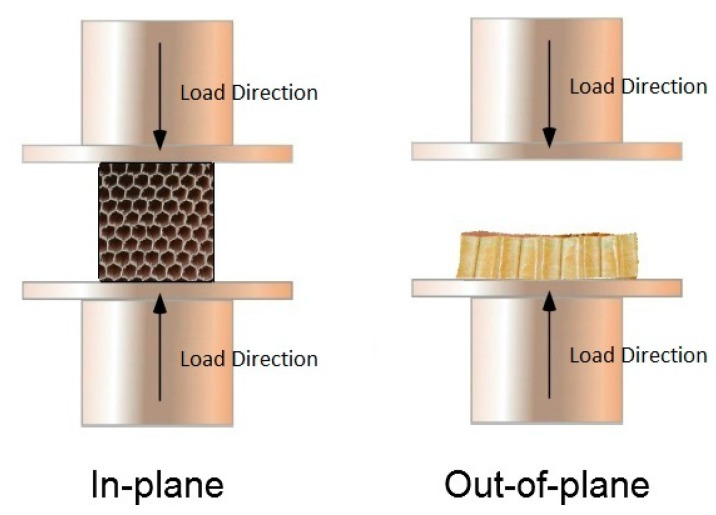
Schematic representation of conducted compression tests.

The mechanical properties of the synthesized composites were tested in-plane and out-of-plane as shown in Figure 12.

**Figure 13:**
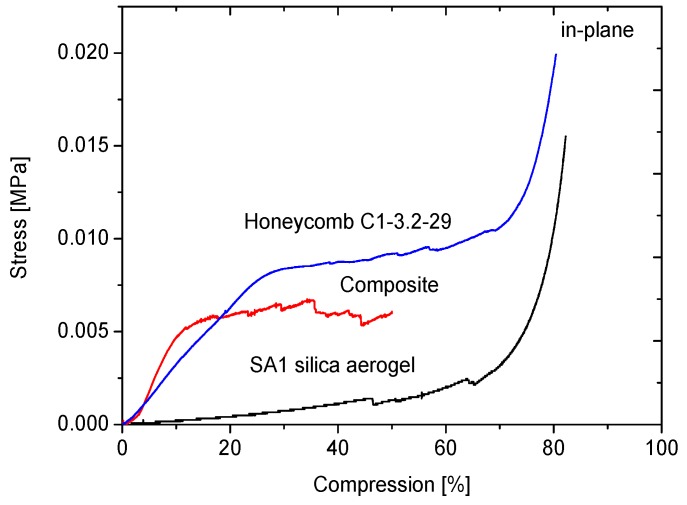
Compression curves of SA1 with C1-3.2-29 in-plane.

**Figure 14:**
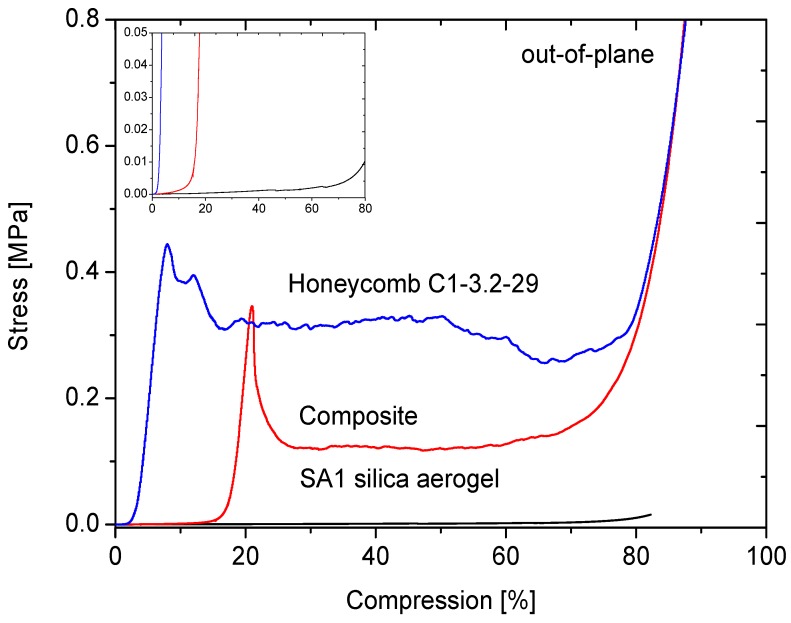
Compression curves of SA1 with C1-3.2-29 out-of-plane.

**Figure 15:**
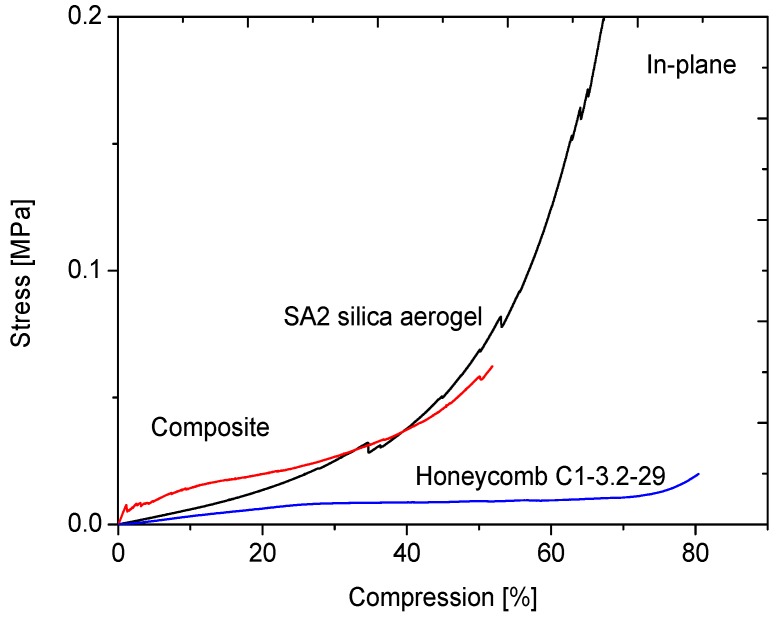
Compression curves of SA2 with C1-3.2-29 in-plane.

**Figure 16:**
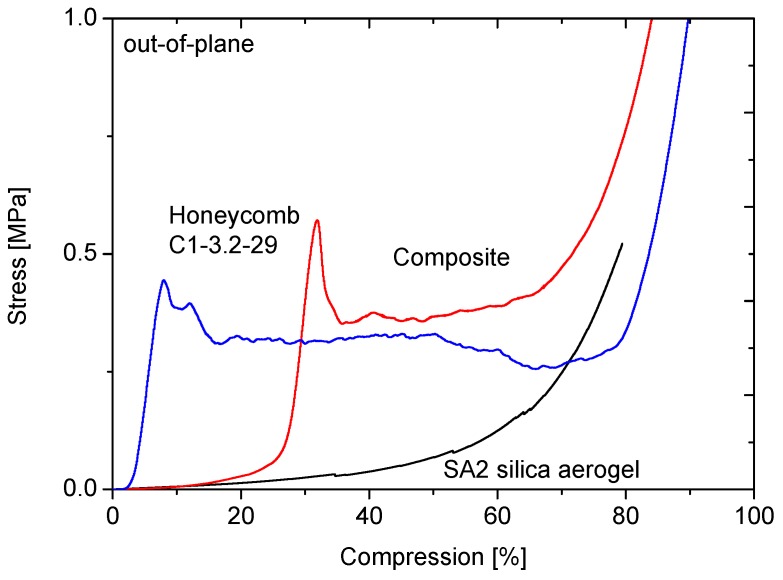
Compression curves of SA2 with C1-3.2-29 out-of-plane.

The effect of the aerogel filling of the honeycombs on the mechanical properties will be discussed on the example of the C1-3.2-29 honeycomb material. Figure 13, Figure 14, Figure 15 and Figure 16 display the load-displacement data of four representative samples of C1-3.2-29. Each figure compares the pure aerogel SA1 or SA2 as references, the empty honeycombs and the composite material.

The soft and super-flexible SA1 silica aerogel was compressed up to 80%. After reaching 40% of compression, a first small crack was observed. As shown in Figure 13, they were followed by several other cracks. They indicate irreversible deformation of the material. Further compression leads to densification of the porous structure. The strain increased rapidly and reached 0.015 MPa at 80% compression.

The stress-compression curve of empty honeycomb in-plane shows three regions. The first region is characterized by a constant slope with rising stress. This slope was used to determine the compressive modulus. After reaching a maximum, the region of elastic deformation ends and a plateau is reached, which indicates the second region. Further deformations in the structure are reflected in the long plateau, which extends up to 70%. Many small cracks are characteristic for the deformations of the walls. Finally, when the cell walls touch each other, densification starts and the stress rises [28].

In contrast, the curve of the composite exhibits a higher slope in the first region. The filling of honeycomb cells increases the deformation resistance, even if the filling material is very soft as, in our case, the SA1 aerogel. The compressive modulus of the honeycomb material is increased by factor 2.3 and compared to the pure aerogel by a factor of ten. The roughness of the curve in the plateau region indicates several large cracks. They reflect a rupture inside the composite material.

Nevertheless, some difficulties occurred during testing. As loading progressed, the samples bent slightly so that perfect uniaxial load could not be reached. Thicker samples could help to avoid this problem.

The stress-compression curves out-of-plane show another progression in the Figure 14. The loading capacity in that direction is much higher. A first deformation seen as a bending of the stiff walls is observed after reaching 0.4 MPa. Then, the resistance becomes weaker and the walls start bending at several positions. After 80% compression, irreversible densification of the honeycomb material takes place.

The curve of the composite material looks quite similar. The delayed rise of the curve for the out-of-plane measurements is caused by protruding aerogel, which could not be cut perfectly without damaging the composites structure, resulting in an offset. For the honeycomb material, the nearly linear behavior of the first part, which can be found for all samples, indicates elastic behavior over a large range of deformation. The linear relation between stress and compression ends with a maximum followed by a region of almost constant stress. In out-of-plane, the compressive modulus decreased by 5%. The weakening of the composite could be caused by defects in the material. As shown in Figure 7, air bubbles were formed between cell walls and aerogel, so that a continuous contact is not given in the composite. One can assume that, under loading, the cracks will start at these positions.

**Figure 17:**
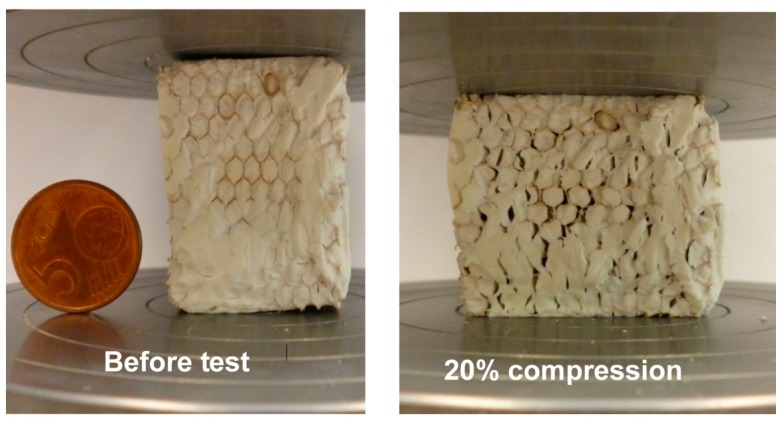
Compression test of SA2 with C1-3.2-29 honeycombs in-plane. Several cracks and holes arose during compression.

The compression test of SA2 aerogel is shown in Figure 15. A short linear region at the beginning, where the compression was determined, is followed by two jumps in the curve. Compared to SA1, we can see a steeper slope in the Hookean region, which speaks for a higher compressive modulus. When the compression of 30% is exceeded, irreversible deformations occur in the aerogel material. After first fracturing of pore walls, which is reflected in the jumps, the stress starts to rise. The aerogel loses porosity and becomes a compact material. The compressive modulus of the composite material is four times higher than that of the empty honeycomb material itself. Under in-plane loading, several cracks along the walls arise. As seen in Figure 17, the contact between the two materials of the composite under uniaxial load is the weakest point. To improve the strength of adhesion, a pre-treatment of the surface of the honeycomb with e.g., surfactants, should be performed.

The out-of-plane compression curves of the composite materials containing SA2 show similar behavior as the ones with SA1 aerogel. The stiffness of these composites is improved by 13%.

**Figure 18:**
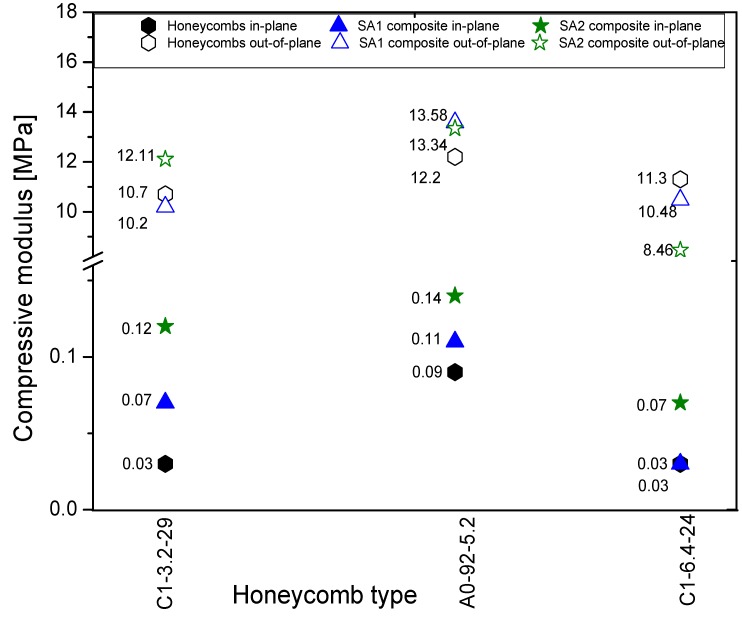
Compressive moduli of empty and field honeycombs in two directions. Since we could observe defects in the manufactured materials (air bubbles, holes), these weak points should be prevented. Research along this line is currently in progress.

The compression curves of all other composites produced, combining the two types of aerogel SA1 and SA2 with various honeycomb materials, look similar to the given examples. The results of the compression tests are summarized in Figure 18. In all cases, the in-plane compressive moduli of the composite materials are increased. Due to higher stiffness of SA2 aerogel, the corresponding composite materials are stiffer too. The highest values are reached with the medium cell size A10-92-5.2 honeycomb from HEXEL^®^. Honeycombs of type C1-6.4-24 with bigger cell size and therefore highest aerogel amount showed the lowest stiffness.

The results of the out-of-plane compression tests are similar. The highest improvement is carried out using A10-92-5.2 honeycomb material with a cell size of 6.4 mm. No increase is reached for both types of aerogel. In general, weakening of a honeycomb material is caused by the high capability of moisture absorption of aramid [29]. The cells of the honeycomb materials consist of aramid fibers, which take up humidity by capillary forces. For honeycomb materials with cell size of 3.2 mm, the moisture absorption is 1.3% and, for 4.8 mm, it is 1.7% [20]. Therefore, to avoid moisture absorption, all honeycomb materials of aramid are covered by phenolic resins. As soon as the honeycombs are cut, the protecting layer is broken and moisture can be absorbed via the cut surfaces.

To summarize the thermal and mechanical properties of the silica-aerogel aramid honeycomb composite materials, we point out that the lowest thermal conductivity could be reached with SA1 aerogels and C1-6.4-24 honeycomb materials. In contrast, the same composite possesses poor mechanical properties. The highest improvement in terms of stiffness could be achieved with SA2 and SA1 aerogels and A10-92-5.2 type honeycomb materials. The honeycombs A10-92-5.2 possess the highest strength (0.09 MPa in-plane and 12.2 MPa out-of-plane) compared with other honeycomb materials. It can be expected that the combining of A10-92-5.2 with aerogels would lead to highest values. On the other side, the ratio of both materials in the composite plays also an important role. The hybrid with the largest cell size (C1-6.4-24) contains a high amount of soft aerogel, which reduces the compressive strength. Obviously, the cell size of A10-92-5.2 represents the best ratio of combined materials.

## 3. Conclusions

Two different silica aerogels, filled in the cells of aramid honeycomb structures of different cell dimensions, are synthesized and investigated. Different types of aerogels: super-flexible SA1 and low-flexible SA2 are successfully combined with the honeycomb material and suitable adherence and thermal conductivities of 0.036–0.044 W·(m·K)^−1^ are achieved. Both thermal conductivity and compressive modulus depend on cell dimension of honeycombs. High aerogel volume fractions lead to the highest decrease of thermal conductivity but not to an improvement of the mechanical properties. The mechanical properties on the other hand are remarkably increased, compared to pure aerogels, and represented by stress-strain curves generated from uniaxial compression tests. The huge differences in mechanical properties are caused by the different microstructures of the aerogels as observed in SEM.

These results complement other experimental advances in the investigation of aerogel-honeycomb-composite material and provide a better understanding of the interaction of the aerogels tested both in synthesis, under uniaxial loading and with respect to thermal conductivity. Depending on the intended application, a careful choice of the utilized honeycomb material will give the opportunity to tailor the composite materials characteristics. It was demonstrated that aerogel-honeycomb-composite materials have the potential to enable new practical applications for silica aerogel insulation via diminishing the aerogels limiting fragility.

Finally, a non-toxic, non-fuming, flame retarding light insulation material with sufficient contact between the two composite materials has been presented, which shows drastically improved mechanical properties in contrast to pure aerogel while maintaining low thermal conductivity.

## 4. Experimental Section

### 4.1. Materials for Synthesis

Methyltrimethoxysilane (MTMS) 95% was purchased from Sigma-Aldrich. Tetraethyl orthosilicate (TEOS) > 99%, [3-(2,3-Epoxypropoxy)-propyl]-trimethoxysilan (GPTMS) ≥ 97%, ammonia (NH_4_OH) 28%–30%, and hydrochloric acid 10^−4^ M from Merck. The surfactant *N*-(Hexadecyl)trimethyl-ammonium chloride (CTAC) 96% and diethylentriamine (DETA) 99% were supplied by Alfa Aesar. Solvents ethanol 96% and methanol 98.5% were purchased by Walter CMP and VWR International, respectively. Deionized water was used for synthesis. Carbon dioxide 4.5 (purity ≥ 99.995%) for supercritical drying was purchased by Praxair, Germany. Sealable polypropylene containers of 60 mL for gelation (with screw top) and 400 mL containers for washing (press-on lid) were purchased from VWR, Germany. The chemicals were used as received.

The honeycombs with properties given in Table 3 were purchased by Hexcel and Schütz Industry Services.

**Table 3:** Tabular listing of the aerospace qualified honeycomb used [20,21].

Manufacturer	Utilized Honeycomb	Cell Size (mm)	Height (mm)
Schütz Industry Services	C1-3.2-29	3.2	10
Schütz Industry Services	C1-6.4-24	6.4	10
Hexcel	A10-92-5.2	5.2	10

### 4.2. Synthesis of Aerogels-Honeycomb Composites

The aramid honeycombs were first cut to rectangular samples of 35 × 35 mm and 10 mm height. They were placed on the bottom of sealable polypropylene containers.

For this study, two types of silica aerogel were synthesized. We used several precursors and alkaline catalysts to achieve different mechanical properties of aerogels.

Synthesis of MTMS based silica aerogel: for the synthesis of MTMS based silica aerogel, the molar ratios of MTMS:Methanol:CTAC:NH_4_OH:HCl were set to 1:35:4:4:4. In the synthesis, the precursor MTMS and methanol as solvent were mixed with the surfactant CTAC in a beaker at room temperature and stirred 5 min with a cross magnetic stirring bar. Then, 0.0001 M HCl solution was added to start the hydrolysis. The mixture is then stirred for 3 h with the same stirring velocity while being covered with aluminum foil. After 3 h, ammonia as an alkaline catalyst was added to start the condensation reaction and stirred for a few seconds and transferred into polypropylene beakers of 52 mm diameter with 35 mm × 35 mm honeycomb samples inside. The honeycombs were completely covered with the solution. These beakers are then transferred into an oven for 3 days for gelation and aging at 50 °C.

Synthesis of MTMS-GPTMS based silica aerogel: The same procedure is used for the low-flexible aerogel SA2, but with a molar ratios of MTMS:GPTMS:Methanol:CTAC:DETA:HCl of 1:0.25:30:0.071:0.125:30 with added GPTMS and alkaline DETA instead of NH_4_OH. The synthesis of this aerogel is based on the work of Aravind *et al.* [30].

After aging, the gels were cooled down to the room temperature and were transferred in an ethanol bath to remove the residual chemicals and to exchange water with ethanol. The ethanol was refreshed twice a day and six times in total, which ensures that water in the samples is exchanged with ethanol, which is soluble in supercritical carbon dioxide used in the final supercritical drying process. The supercritical drying was carried out for 32 h and with CO_2_ in an autoclave at 46 °C and 97 bars with a mass flow rate of 14 kg·h^−1^. The degassing rate was adjusted to 0.1 bars per minute. Finally, cylindrically shaped aerogel-honeycomb-composite samples are obtained, which then are carefully cut into 35 mm × 35 mm quadratic samples with height of 10 mm.

### 4.3. Characterization

Since the aim of this work is improvement of thermal and mechanical properties, the analysis was focused on these two aspects. The thermal conductivity was measured via Transient Plane Source (TPS) method using a Hot Disk Thermal Constants Analyzer TPS2500 (HotDisk, Göteborg, Sweden) [31,32]. The measurements were done between at 23.6–27.9 °C and 1003–1015 bar atmospheric pressure, with humidity ranging between 43.8% and 67.9%. The compression tests were done at ambient conditions with help of a compression machine (Latzke, Wiehl-Marienhagen Germany) and load cells of 5000 N, with 1 mm·min^−1^ speed of compression. Since standard testing methods for aerogels do not exist, the compression tests were based on recommendation of ISO 844:2014. The samples were compressed up to 50% of their original length. These data was enriched with complementing data of envelope density and microstructure, which characterize the aerogels themselves. The envelope density of the samples was measured pycnometrically with a GeoPyc 1360 (Micromeritics, Norcross, GA, USA). The skeletal density was measured with AccuPyc (Micromeritics, Norcross, GA, USA). Surface area of aerogels was determined by nitrogen adsorption-desorption method BET (TriStarII, Micromeritics, Norcross, GA, USA).The microstructure of the aerogels and composites, especially the bonding between the aramid honeycomb and the aerogel, was studied with the help of SEM (Merlin, Carl Zeiss SMT, Oberkochen, Germany), using the detector for secondary electrons.
